# *RAS* testing in metastatic colorectal cancer: advances in Europe

**DOI:** 10.1007/s00428-015-1876-7

**Published:** 2015-11-16

**Authors:** J Han JM Van Krieken, Etienne Rouleau, Marjolijn J. L. Ligtenberg, Nicola Normanno, Scott D. Patterson, Andreas Jung

**Affiliations:** Department of Pathology, Radboud University Medical Center, P.O. Box 9101, 6500 HB Nijmegen, Netherlands; Curie Institute, Paris, France; Cell Biology and Biotherapy Unit, INT-Fondazione Pascale, Naples, Italy; Amgen Inc., Thousand Oaks, CA, USA; Institute of Pathology, University of Munich, Munich, Germany; German Cancer Consortium (DKTK), German Cancer Research Center (DKFZ), Heidelberg, Germany; Gilead Sciences, Inc., Foster City, CA USA

**Keywords:** Colorectal cancer, *RAS*, Biomarkers, Sequencing, Quality assurance programs

## Abstract

Personalized medicine shows promise for maximizing efficacy and minimizing toxicity of anti-cancer treatment. *KRAS* exon 2 mutations are predictive of resistance to epidermal growth factor receptor-directed monoclonal antibodies in patients with metastatic colorectal cancer. Recent studies have shown that broader *RAS* testing (*KRAS* and *NRAS*) is needed to select patients for treatment. While Sanger sequencing is still used, approaches based on various methodologies are available. Few CE-approved kits, however, detect the full spectrum of *RAS* mutations. More recently, “next-generation” sequencing has been developed for research use, including parallel semiconductor sequencing and reversible termination. These techniques have high technical sensitivities for detecting mutations, although the ideal threshold is currently unknown. Finally, liquid biopsy has the potential to become an additional tool to assess tumor-derived DNA. For accurate and timely *RAS* testing, appropriate sampling and prompt delivery of material is critical. Processes to ensure efficient turnaround from sample request to *RAS* evaluation must be implemented so that patients receive the most appropriate treatment. Given the variety of methodologies, external quality assurance programs are important to ensure a high standard of *RAS* testing. Here, we review technical and practical aspects of *RAS* testing for pathologists working with metastatic colorectal cancer tumor samples. The extension of markers from *KRAS* to *RAS* testing is the new paradigm for biomarker testing in colorectal cancer.

## Introduction

Personalized medicine and tailoring of therapy to individual patients is a promising approach for maximizing efficacy and minimizing the toxicity of anti-cancer treatment [1]. Furthermore, such an approach can generate healthcare cost savings, as treatments are given only to patients likely to benefit [2]. In recent years, a few molecular tumor biomarkers—characteristics “that can be objectively measured and evaluated as an indicator of pathogenic processes or treatment response” [3]—have been identified, enabling anti-cancer treatments to be better tailored to individual patients’ tumors [1]. Those molecular biomarkers have provided alternative therapeutic options and improved patient outcomes, especially in metastatic colorectal cancer (mCRC). In the European Union, there were an estimated 342,000 new cases of colorectal cancer in 2012 (46.3 per 100,000 individuals), and mCRC remains the second leading cause of cancer-related deaths (215,000 deaths in 2012) [4].

Epidermal growth factor receptor (EGFR)-targeted monoclonal antibodies (mAbs) have been investigated for the treatment of mCRC, but the benefits to patients were small in initial clinical trials when these agents were unselectively added to standard care. Subsequently, *KRAS* mutations, particularly those in exon 2 (detectable in about 30–40 % of patients with mCRC) [5, 6] were identified as a predictive biomarker of resistance to the EGFR-targeted antibodies. These mutations result in constitutive activation of the GTPase *KRAS*, leading to activation of signaling downstream of the EGFR [7–10]. Consequently, in patients with exon 2 wild-type (WT) *KRAS* tumors, addition of EGFR inhibitory antibodies to standard chemotherapy significantly improved progression-free survival (PFS) versus chemotherapy alone [8, 11]. For example, in the phase III, first-line PRIME study, oxaliplatin, 5-fluorouracil (5-FU), and leucovorin (FOLFOX4) plus panitumumab was associated with a median PFS of 9.6 versus 8.0 months for FOLFOX4 alone (*p* = 0.02), while median overall survival [OS] was 23.9 and 19.7 months, respectively (*p* = 0.072) [8]. Similarly, median PFS with irinotecan, 5-FU, and leucovorin (FOLFIRI) plus cetuximab in the CRYSTAL study was 9.9 versus 8.4 months with FOLFIRI alone (*p* = 0.0012) and OS was 23.5 versus 20.0 months (*p* = 0.0093), respectively [11]. In patients with mutant *KRAS* tumors, combination of anti-EGFR therapy with irinotecan-based chemotherapy is associated with little or no benefit [9, 12, 13], while combination of anti-EGFR therapy with oxaliplatin-based chemotherapy in such patients may be detrimental to both PFS and OS [8, 14, 15].

While *KRAS* exon 2 mutations are undoubtedly useful for predicting a lack of activity of EGFR-targeted mAbs, use of this biomarker increased response rates only from ~15 % in an unselected population to ~30 % in patients with *KRAS* exon 2 WT mCRC [16]. Analyses of tumor samples from the PRIME study in mCRC have shown that more comprehensive *RAS* testing (i.e., exon 2, 3, and 4 of both *KRAS* and *NRAS*) better selects those patients more likely to respond to EGFR inhibitors, with *RAS* WT populations experiencing a statistically significant improvement in OS versus chemotherapy alone (26 versus 20.2 months; *p* = 0.04) [14]. Furthermore, data from the phase II PEAK and phase III FIRE-3 studies indicated that patients with WT *RAS* tumors experienced greater benefit from mFOLFOX6 plus panitumumab (PEAK) or FOLFIRI plus cetuximab (FIRE-3) than from mFOLFOX6 or FOLFIRI, respectively, plus bevacizumab in terms of PFS in PEAK only (PEAK, 13.0 versus 9.5 months; *p* = 0.029 and FIRE-3, 10.0 versus 10.3 months; *p* = 0.55) and OS in both (PEAK, 34.2 versus 24.3 months; *p* = 0.009 and FIRE-3, 28.7 versus 25.0 months; *p* = 0.017) [17, 18]. Taken together, mutations in either *KRAS* or the closely related *NRAS* gene, but not in the *BRAF* gene, were found to be associated with lack of response to EGFR-targeted mAbs [18–24].

In several large studies including patients with mCRC, over half of patients were found to have tumors harboring mutations in *KRAS* or *NRAS* (Table 1) [10, 14, 18, 19, 25–31]. For example, 17 % of patients with tumors WT for *KRAS* codons 12 and 13 in the PRIME study (~10 % of patients overall) were found to have mutations in other *KRAS* codons and/or *NRAS* [14]. A pooled analysis of five studies (*n* = 2832 patients with *KRAS* and *NRAS* data available) showed that the prevalence of *RAS* mutations was 55.9 % (95 % confidence interval [CI], 53.9–57.9 %) [32]. More recently, a meta-analysis of nine randomized controlled trials (*n* = 5948) showed that EGFR-targeted mAb therapy in a *RAS* WT population had a significantly superior PFS (*p* < 0.001) and OS (*p* = 0.008) treatment effect compared with in a *RAS* mutant population [33].

**Table 1:** Prevalence of *RAS* mutations in patients with metastatic colorectal carcinoma

Study	Detection method	*RAS* mutation prevalence						
		*KRAS*			*NRAS*			Total
		Exon 2	Exon 3	Exon 4	Exon 2	Exon 3	Exon 4	
PRIME [14]	TheraScreen	40 %	4 %^a^	6 %	3 %	4 %^a^	0 %	52 %^a^
20050408 [19]	DxS/Qiagen/NGS/Sanger	42 %	2 %^a^	NS	5 %^a^		NS	NS
20050408 (updated) [26]	NGS/Sanger + WAVE/SURVEYOR®	43 %	5 %^a^	5 %	4 %	3 %^a^	1 %	NS
20050181 [25]	DxS/Qiagen/NGS/Sanger	45 %	4 %	8 %	2 %	6 %	0 %	58 %
PICCOLO [27]	Pyrosequencing	NA^b^	NA^b^	4 %^c^	6 %^a^		NS	NS
PEAK [18]	Therascreen + WAVE/SURVEYOR®	NA^d^	4 %	7 %	5 %	6 %	0 %	NS
CRYSTAL [10]	LightMix	36 %	NS	NS	NS	NS	NS	NS
CRYSTAL (updated) [28]	BEAMing	37 %	3 %	5 %	4 %	3 %	2 %	43 %
COIN [29]	Pyrosequencing	43 %^a^		NS	4 %^a,e^		NS	NS
FIRE-3 [30]	Pyrosequencing	NA^d^	4 %	5 %	4 %	2 %	0 %	NS
OPUS [31]	BEAMing	NA^d^	6 %	9 %	7 %	5 %	1 %	NS

*BEAM* beads, emulsions, amplification, and magnetics, *NGS* next-generation sequencing, *NS* not specified, *PCR* polymerase chain reaction

^a^Not including codon 59

^b^Study population limited to patients with *KRAS* (exon 2 and exon 3 codon 61) wild-type tumors

^c^Not including codon 117

^d^Study population limited to patients with *KRAS* (exon 2) wild-type tumors

^e^Not including codon 13

As patients with mutant *RAS* tumors do not benefit from EGFR inhibitors, and may even experience worse outcomes when EGFR inhibitors are combined with oxaliplatin-based chemotherapy [14, 15], the European Public Assessment Report summaries of product characteristics (SmPCs) for panitumumab and cetuximab specify that “evidence of wild-type *RAS* (*KRAS* and *NRAS*) status is required before initiating treatment” [34, 35]. *RAS* gene testing has therefore become an important part of the work-up of patients with mCRC in Europe. Thus, it is essential that genetic testing is conducted to a high standard and as quickly as possible in case a patient is eligible for EGFR-targeted treatment. The highest quality of testing is achieved when the genetic analysis is carried out in the context of a histopathological evaluation [36, 37] to ensure that appropriate tissue areas are included in the analysis. To test and ensure the quality of *RAS* testing (i.e., the correct identification of WT [specificity] and mutant *RAS* [sensitivity] to eliminate false-positive and false-negative results), external quality assurance (EQA) programs play a critical role. This view is supported by the European Medicines Agency (EMA), as the SmPCs for panitumumab and cetuximab state that “mutational status should be determined by an experienced laboratory using validated test methods for detection of *KRAS* and *NRAS* (exons 2, 3, and 4) mutations” [34, 35]. As “experience” is only vaguely definable, a test by an EQA program is a suitably objective tool to verify a laboratory’s experience as noted in the SmPC for panitumumab: “if [panitumumab] is to be used in combination with FOLFOX then it is recommended that mutational status be determined by a laboratory that participates in a *RAS* EQA program or WT status be confirmed in a duplicate test” [34].

Full tumor *RAS* testing—rather than solely *KRAS* exon 2 testing—is included in treatment guidelines for mCRC such as those from the European Society for Medical Oncology (ESMO), the National Comprehensive Cancer Network (NCCN), the German Cancer Society Association of Medical Oncology (AIO), and the Dutch Landelijke Werkgroep Gastro Intestinale Tumoren [38–41].

The shift from screening a single *KRAS* locus to multiple *RAS* loci highlights the evolution of biomarker testing to detect multiple mutation sites. This change in approach may affect the choice of screening methods used, as many different techniques for assessing *RAS* mutation status are now available [42]. The aim of this review is to describe these different methods, focusing on those most commonly used in Europe: sequencing (Sanger sequencing, pyrosequencing, next-generation sequencing [NGS] techniques), other in vitro diagnostic techniques, and laboratory-developed techniques. These approaches differ in both their limit of detection (LOD; sensitivity) and their specificity. Some of the key issues in *RAS* testing will be discussed, including sensitivity of testing and EQA programs.

### Initial material—DNA from formalin-fixed, paraffin-embedded tissue blocks

To assess *RAS* mutation status, analysis is usually performed on DNA extracted from formalin-fixed, paraffin-embedded (FFPE) tissue blocks [43]. As a result of chemical modification and DNA fragmentation, the amplicon size in polymerase chain reactions (PCR) is limited to about 150 base pairs, when sequencing is performed on FFPE-derived DNA [44]. Moreover, for NGS, the initial procedure for DNA isolation from FFPE tissue blocks remains the same as for conventional DNA sequencing [43] and is, therefore, subject to some of the same issues of DNA quantity and quality.

### DNA sequencing methods

Most sequencing methods can screen a gene locus for mutations. Newer sequencing methods provide quantitative information and increase the number of loci that can be analyzed in parallel.

Sanger sequencing (also known as “terminated chain sequencing” or “dideoxy sequencing”) is considered to be a standard method for analyzing DNA sequences and thus also for *RAS* testing [43, 44]. Overall, it is the most widely used method of *RAS* testing in Europe [45], although its use varies from country to country. Major improvements to the original Sanger technique have resulted in reduced reagent volumes and consumable costs, as well as increased throughput [46], although it is still considered time consuming compared with some other methods [44]. The advantage of this method is its ability to detect all types of mutation (single-nucleotide substitutions, insertions, and deletions) in the amplified locus. If DNA is isolated from FFPE tissue, the size of the amplicons to be analyzed should not exceed 150 base pairs. To ensure reproducibility, bidirectional sequencing reactions are performed using forward and reverse primers [47]. Each run gives the nucleotide sequence of one amplified locus; up to 96 runs can be performed in parallel (usually 16 to 24). In addition, the LOD for Sanger sequencing is relatively modest compared with other techniques. Multiple studies have shown that Sanger sequencing requires at least 10–25 % of the neoplastic cells in the sample to contain *KRAS* mutations for reliable detection [48]. Several enrichment methods are available, however, to increase the concentration of mutant DNA, thus improving the overall sensitivity of the detection process (Table 2) [49–53]. Another limitation of Sanger sequencing is the low level of precision in the variant fraction (i.e., the percentage of tested alleles that are mutant); the error rate (i.e., the percentage of incorrectly identified bases) of the Sanger sequencing reaction is estimated at 0.001 to >1 %, depending on the software that is used post processing [54].

**Table 2:** Methods for enriching mutant DNA for *RAS* testing [49–53]

Method	Features
Microdissection	Allows precise analysis of tumor tissue by reducing contamination with normal tissue and, therefore, improving sensitivity for *RAS* mutations present in the neoplastic cell sample
COLD PCR	Exploits the critical temperature at which mutation-containing DNA is preferentially melted over WT (sensitivity, 0.01–10 %)
Allele-specific amplification	Uses primers designed with a 3′ terminal nucleotide that pairs with the mutant sequence but not with the WT (e.g., the amplification refractory mutation system) (sensitivity, 0.1–1 %)
Blocking amplification of WT sequence	For example, by binding high-affinity peptide nucleic acid probes (PCR clamp; sensitivity in routine applications, 0.1–1 %)
Selective destruction of WT samples	Exploiting restriction enzyme sites in the WT sequence (e.g., restriction endonuclease-mediated selective PCR and simultaneous PCR/restriction fragment length polymorphism) (sensitivity, 0.001–0.1 %)

*COLD* co-amplification at lower denaturation temperatures, *PCR* polymerase chain reaction, *WT* wild-type

Pyrosequencing is an alternative method to Sanger sequencing that can provide both quantitative information and a low technical LOD (5–10 %) [55], although the sequencing error rate may be higher (4–25 %) [56]. During pyrosequencing, the incorporation of each nucleotide is followed in real time by the emission of a specific light unit based on the amount of pyrophosphate, which is released in stoichiometric amounts during the elongation reaction and converted by an enzymatic cascade into adenosine triphosphate (ATP) molecules each time a nucleotide is incorporated. The ATP serves as the energy donor in a bioluminescence reaction resulting in emission of light that can be measured as a peak in the pyrogram output. Pyrosequencing allows the local separation of mutant and WT signals, although the presence of homopolymers can limit the method, as linearity is present only for some nucleotides. In this approach, each run gives the nucleotide sequence of one amplified locus, but the size of the sequence (less than 20 base pairs) is often shorter than those produced via Sanger sequencing. Up to 96 runs can be performed in parallel.

### Next-generation DNA sequencing

NGS platforms involve massively parallel sequencing of bulks of clonally amplified DNA molecules that are spatially separated and have helped to shift the focus of sequence analysis from a few loci to multiple genes and loci. With NGS, each run can give the nucleotide sequence for several loci and several tumor samples. Importantly, NGS methods can detect any variant from single-nucleotide changes to insertions or deletions, and even translocations, in a single multiplex PCR reaction. Depending on the depth of sequencing, the LOD of NGS is about 1–5 %. Results are provided as quantitative information. The most sensitive part of the NGS is the bioinformatic pipeline generating Binary Sequence Alignment Map/Sequence Alignment Map format (BAM/SAM) files. Specific statistical algorithms are used to score the quality of a single read by aligning the sequence to a reference genome. As a consequence, some readouts do not detect all of the deletions, insertions, or other mutations that are detectable with Sanger sequencing, as some mutations are found by the basic algorithms but subsequently excluded and, therefore, do not occur in the resulting variant caller files. Therefore, extensive validation of the entire procedure is essential, as is the use of internal control to assess the reproducibility of the runs.

With the increasing number of biomarkers, NGS allows screening of a large number of patients and markers with a fixed and limited amount of DNA. For NGS using Ion Torrent technology, as little as 1 ng input material is needed, whereas conventional sequencing technologies need 10 to 20 ng per run and marker [57]. As a first step, a DNA library is generated by amplification of the DNA fragments to be sequenced (the gene panel) in a single reaction (sometimes in up to five different tubes) or capture hybridization followed by the amplification of this pre-library either by emulsion PCR (emPCR; e.g., on spheres) or by bridge PCR on solid flow cells [46]. As capture hybridization needs much more input material compared with the panel-amplification approach, the latter is mostly used in the analysis of biomarkers. Ultimately, a single NGS run may comprise many millions or even billions of spatially distributed, clonally amplified amplicons [58]. Custom assays can be developed (AmpliSeq™ for Ion Torrent, GeneRead for Qiagen, TruSeq™ for Illumina) using amplicons designed with a starting size of 150 bp, optimal for FFPE samples. Finally, the quality of the sample can be extremely limited, and strategies have been proposed to identify samples with low probability of success (e.g., the Illumina FFPE QC Kit based on qPCR validation) before any NGS run. This highlights that not all samples are suitable for analysis on these platforms.

Historically, three principal NGS technologies have been commercially available [58]. The first of these, sequencing-by-ligation, no longer has any clinical application. The second is sequencing-by-synthesis [59], and the third is based on the pyrosequencing approach. As an alternative to pyrophosphate measurement, hydrogen ions/protons can be used as a hydrogen ion is released for each nucleotide incorporated. These can be measured as an induced currency peak on a negatively charged semiconductor, which is the basis of the detection unit or chip. Currently available NGS platforms are summarized in Table 3.[60–62] Of these, the Illumina MiSeq and Life Technologies’ Ion Torrent Personal Genome Machine™ (PGM™) are the most commonly used in Europe (H. Van Krieken, personal communication), and NGS sequencing methods are expected to replace older methods completely in the coming years.

**Table 3:** Examples of currently available next-generation sequencing platforms

	Principle	Amplification
454 (Roche) [60]	Pyrosequencing	Emulsion PCR
Junior (Roche)	Pyrosequencing	Emulsion PCR
HiSeq/MiSeq (Illumina) [61]	Reversible termination	Bridge PCR
Ion Torrent (Life Technologies) [62]	Semiconductor sequencing	Emulsion PCR

*PCR* polymerase chain reaction

#### MiSeq (Illumina Sequencing Systems)

MiSeq is based on a sequencing-by-synthesis approach [59], using fluorescently labeled reversible-terminator nucleotides and clonally amplified DNA templates on acrylamide-coated glass flow cells [63]. Originally, MiSeq was developed as a modification of the earlier Genome Analyzer and HiSeq machines and was designed for lower throughput and fast-turnaround appropriate for smaller laboratories and clinical diagnostics. The MiSeqDx is a special type of MiSeq developed for clinical analysis [63, 64]. The error rate of the Illumina technology is estimated to be <0.4 % [63, 65]. More recently, Illumina has introduced the NextSeq system, which is broadly similar to MiSeq, although the run time is faster (12–30 versus 5–55 h for MiSeq) [61].

#### Ion Torrent (Life Technologies)

The Ion Torrent PGM™ sequencer is based on ion semiconductor technology [66]. DNA fragments are clonally amplified by emulsion PCR on the surface of 3-μm diameter beads, which are then loaded into wells on a proton-sensing silicon wafer. PGM™ was the first commercial sequencing machine that did not require fluorescence and camera scanning, resulting in higher speed, lower cost, and smaller instrument size [64]. The error rate with Ion Torrent sequencing has been estimated at 1.8–1.9 % [63, 65], mostly because of higher error rates in the detection of homopolymer stretches. Ion Torrent sequencing has been recently used to assess *RAS* status of colorectal carcinoma tumors in the CAPRI-GOIM clinical trial [57], and a panel of well-known predictive markers in the receptor tyrosine kinase pathway, including *KRAS* and *NRAS*, has been developed by the OncoNetwork Consortium [67].

### Hypersensitive methods

For the detection of single mutation events in a background of WT sequences, digital PCR techniques [68] were developed that can achieve LODs of <0.1 % for specific hotspots. If the coverage, and thus the depth, of NGS techniques are increased, they can also reach similar LODs [69]. Sensitivity can also be increased by using single-molecule techniques that discriminate between biological and technical duplicates, such as single-molecule molecular inversion probes [70].

The potential applications for hypersensitive methods include detection of mutations in circulating plasma DNA (the so-called liquid biopsy), monitoring of metastatic disease under therapy, detection of emergence of resistance to targeted agents, analysis of small or poor-quality DNA samples, and as a possible additional parameter alongside histopathology for the staging of tumors [71]. Such approaches have been applied in research and for free plasma DNA detection; there will be no direct application for molecular *RAS* testing in routine clinical practice until the clinical validity of these techniques can be determined. The preparation of such assays is generally easier than for NGS assays, but the scope of the targeted markers is greatly reduced, mostly focusing on a single gene locus or small number of loci.

#### BEAMing

BEAMing (beads, emulsions, amplification, and magnetics) [72] has been used for *RAS* analysis of samples from clinical trials of EGFR inhibitors [73, 74]. Single DNA molecules are attached to single magnetic beads and are amplified by using emPCR, resulting in beads spiked with thousands of clonally identical PCR products. These are hybridized with mutation-specific probes coupled to different fluorochromes, which are then counted by fluorescence-activated cell sorting (FACS). Overall, BEAMing is more suited to the detection of fewer hotspot variants and has the disadvantage that only limited information can be retrieved, which depends mostly on the amount of available fluorochromes and the filter sets of the FACS device. In most cases, up to four different events can be analyzed per run, which limits the width of the detection. A single nucleotide can be analyzed per test (e.g., the nucleotide 34 position in *KRAS*); therefore, for detection of mutations in positions 34, 35, and 38, three BEAMing reactions are necessary. Detection of all three positions (34, 35, and 38) in a single BEAMing reaction can be achieved only by using one fluorochrome each for the WT sequence at nucleotides 34, 35, and 38, and at the expense of knowing the exact type of alteration (e.g., c.34G>A). Although the sensitivity of BEAMing is 0.01 %, it must be emphasized that a cutoff of 5 % mutant alleles in tumor samples has been adopted when this technology has been used in clinical trials [73, 74].

#### Droplet Digital PCR

Droplet Digital PCR (ddPCR) techniques involve compartmentalization of DNA, again using emPCR. Clonally amplified PCR products of an original single DNA fragment are contained in the micelles of a water-in-oil emulsion [75, 76], together with mutation-specific probes that can be obtained from TaqMan® assays (Applied Biosystems). These droplets are counted directly by flow cytometry. This approach can be multiplexed (up to seven in a single run) so, as with BEAMing, multiple runs are necessary to cover the multiple genetic variations of *KRAS* and *NRAS*. The ddPCR detection unit is, however, easier to work with than the BEAMing unit as the ddPCR products can be measured directly without the additional steps required when using the BEAMing technique. ddPCR techniques in association with liquid biopsies show considerable potential, although their precise clinical role is currently unclear [71].

### Approved in vitro diagnostic tools for *RAS* testing

Commercially available kits, instruments, and reagents that are intended by the manufacturer to be used for in vitro examination of patient specimens for the purpose of providing information are governed by European Directive 98/79/EC on in vitro medical devices [77]. Key objectives of this directive are to ensure that in vitro diagnostic medical devices provide a high level of health protection and attain the attributed performance level, and products that conform to this standard carry “CE” marking. The directive does not regulate products for research-use only nor does it cover non-commercialized patient diagnostic tools produced within health institution laboratories for use solely within that environment. It is important to note that the CE mark simply means that the product itself has consistent quality and does not provide any information about specificity and sensitivity in the lab where the kit is used.

The advantage of commercial CE tests is the validation process that they have undergone. Thus, after the in-house verification of a CE-kit, no additional testing is needed, which is in contrast to non-CE-kits where a change in the batch of materials used requires quality testing of the new material. A list of CE-approved *RAS* tests available in Europe (complete as of December 2014, to the best of our knowledge) is shown in Table 4 [78–92]. Some techniques identify the somatic mutation directly, while others are screening methods that involve a two-step process for positive samples. In addition, other CE-approved kits are available for assessing *KRAS* exon 2 status (with or without exon 3 codon 61), and laboratories may wish to use these, supplemented with laboratory-developed tests to complete the *RAS* analysis.

**Table 4:** CE-approved *RAS*-testing kits available in Europe

In vitro diagnostic kit	*KRAS* ^a^	*NRAS* ^a^	Mutant enrichment	Detection method	LOD (%)^b^
CRC *RAS*can™ (SURVEYOR®/WAVE®) [78]	Yes	Yes	None	Mismatched heteroduplex cleavage and DHPLC^c^	2–5
CRC *RAS*seq™ [79]	Yes	Yes	None	Sanger sequencing	5–10
Therascreen® *KRAS* and *NRAS* Pyro [80, 81]	Yes	Yes	None	Pyrosequencing	1–7
Agena^d^ OncoCarta™ [82, 83]	Yes	Yes	None	Mass spectrometry	5–10
LightMix® *NRAS* ex2–4 *KRAS* ex4 [90]	Yes	Yes	PCR clamp (LNA)	Melting curve analysis-based	1
Therascreen® *KRAS* RGQ PCR [84]	Yes	No	Allele-specific probes	RT-PCR	1–6
cobas *KRAS* mutation test [85]	Yes	No	PCR clamp (TaqMelt™)	Melting curve analysis-based	5
Randox biochip array [86]	Yes	No	None	Array hybridization	1
INFINITI® *KRAS* [87]	Yes	No	None	Array hybridization	NS
*KRAS* StripAssay® [88]	Yes	No	PCR clamp (blocker)	Strip hybridization	1
EnteroGen® *KRAS* [89]	Yes	No	Allele-specific probes	RT-PCR	<1
LightMix® *KRAS* codon 12/13 [90]	Yes	No	PCR clamp (LNA)	Melting curve analysis-based	1
PNAClamp™ [91]	Yes	No	PCR clamp (PNA)	RT-PCR	<1
RealQuality RI-*KRAS*MuST [92]	Yes	No	Allele-specific probes	RT-PCR	1

*DHPLC* denaturing high-pressure liquid chromatography, *LNA* locked nucleic acid, *LOD* limit of detection, *NS* not specified, *PCR* polymerase chain reaction, *PNA* peptide nucleic acid, *RT*-*PCR* reverse transcriptase polymerase chain reaction

^a^Methods may not include all exons or relevant codons

^b^Determination methods may vary

^c^Confirmation by DNA sequencing recommended

^d^Formerly Sequenom

Of the available CE-approved kits, only five detect mutations in both the *KRAS* and *NRAS* genes: (1) CRC *RAS*can™ (SURVEYOR®/WAVE®), (2) CRC *RAS*seq™ (both Transgenomics, Inc.), (3) Therascreen® *KRAS* and *NRAS* Pyro® kits (Qiagen), (4) OncoCarta (Agena Bioscience), and (5) cobas® *KRAS* mutation test (Roche) together with LightMix® kits (TIB MOLBIOL) for the cobas system.

CRC *RAS*can™ (SURVEYOR®/WAVE®) uses Transgenomics’ proprietary DNA mismatch-cutting enzyme SURVEYOR® nuclease, with cleavage products separated by denaturing high-pressure liquid chromatography using the WAVE® platform [77]. The nature of the mutation is then confirmed by Sanger sequencing. CRC *RAS*seq™ uses Transgenomics’ proprietary primer sets for PCR amplification and Sanger sequencing of exons 2, 3, and 4 of the *KRAS* and *NRAS* genes [79]. This method was used to identify *RAS* mutation status in mCRC patients included in the PRIME study [14].

Therascreen® *KRAS* and *NRAS* Pyro® kits are based on pyrosequencing technology and consist of two assays: one for codons 12 and 13 and a second for codon 61 [80, 81]. Qiagen has recently launched a new *RAS* Extension Pyro Kit, covering codons 58/59, 117, and 146 of *KRAS* and *NRAS*, which has recently received CE-marked status, although the LOD of this extension kit is not reported [93].

The Agena Bioscience OncoCarta panel includes 12 *KRAS* and eight *NRAS* mutations [82]. Detection is based on the Agena MassARRAY® system, which uses matrix-assisted laser desorption/ionization–time of flight mass spectrometry [94].

The cobas® system works on the basis of high-resolution melting using different PCR reactions together with mutation-specific probes coupled with different fluorochromes using the real-time PCR cobas 480® (Roche) device. After the generation of PCR products, these are melted in a temperature gradient. The melting point of the mutation-specific probe indicates the type of mutation, being specific for either the codon (cobas® *KRAS* mutation test) or the position (LightMix® kits).

The choice of CE-marked *RAS* mutation test by individual laboratories is largely dependent on the available equipment, experience, and costs of the test [95]. The rapid change in knowledge of biomarkers and loci implies that development of additional commercial kits is necessary, and the situation for *RAS* screening, with only five available kits covering all loci, highlights the relatively low reactivity of the market.

### Laboratory-developed methods

Many laboratory-developed methods for *RAS* testing are used for in vitro diagnostics, and still more methods exist for research-use only [42]; the latter are beyond the scope of this review. This is especially true for NGS, as commercially available kits are almost inaccessible, particularly those of CE-quality. CE-marked assays for NGS could be the next step to ensure the quality of reagents and primer pools. Initiatives to standardize a primer set, such as the community panel from Life Technologies and the Ion AmpliSeq™ Colon and Lung Cancer Research Panel v2, are underway, although such primer sets are currently for research-use only [57]. Most commonly, Sanger sequencing- and pyroseqencing-based laboratory-developed methods are used for *RAS* testing [43]. In some countries, such as France, allele-specific PCR is very popular (Dequeker et al., manuscript submitted). Here, mutant alleles are selectively amplified, making this technique highly sensitive for the detection of mutations in the *RAS* genes. There are many different variations of this general principle (Table 5) [96–100], which is reflected in the many variations of allele-specific hybridization methods [43]. In general, laboratory-developed methods offer the possibility of more rapid adaptation to new knowledge of biomarkers and loci, although their use requires a high level of experience and quality management within the laboratory. Moreover, mostly as a result of the costs of CE-marked kits, laboratory-developed techniques are very suitable for testing the sensitivity (LOD) and specificity of *RAS* mutation detection, which is important in the maintenance of quality.

**Table 5:** Allele-specific polymerase chain reaction (PCR) methods for *RAS* testing

Amplification refractory mutation system (ARMS) [96]
Restriction endonuclease-mediated selective (REMS) PCR [97]
Fluorescent amplicon generation (FLAG) [98]
Restriction fragment length polymorphisms (RFLP) [99]
Allele-specific ligation detection reaction [100]

### Sensitivity of *RAS* testing

The sensitivity of mutation detection is influenced by multiple factors, mostly LOD and the minimum amount of required template. The LOD is dependent on the technique used and might therefore be considered as the technical sensitivity, which is defined by the ratio of the measurement signal (mutation) to the background signal (mutant signal in a WT sample) [43]. The sensitivities of available tests range from <1 to 10 % for CE-approved testing methods (Table 4). For laboratory methods, the most sensitive techniques are allele-specific PCR and hybridization assays (0.1–1 %) and gel electrophoresis (e.g., temporal temperature gradient or constant denaturant capillary electrophoresis) (1 %) [43]. Pyrosequencing (5 %), high-resolution melting curve analysis (~10 %), and Sanger sequencing (10–25 %) have a lower level of technical sensitivity [43, 101]. This highlights the benefit of histopathology as the basis of mutation detection: sensitivity can be controlled, as the total amount of tissue—reflecting the amount of template, and relative fraction of neoplastic cells within the tumor (measured as area or total nuclei)—reflecting the LOD, can be determined (semi) quantitatively. For example, a *KRAS* mutation in a small metastatic focus of neoplastic cells in a lymph node can escape detection when entire tissue sections of the lymph node are used for DNA isolation (i.e., normal cell DNA content is far greater than neoplastic cell DNA). After careful microdissection of the neoplastic cells, however, the *KRAS* mutation can unequivocally be identified. Thus, in clinical practice, the sensitivity of the entire process must be considered, not just the sensitivity of the technique being used.

Unfortunately, the sensitivity of mutation detection—which needs to be related to the clinical effect—in the reference studies establishing the validity of *RAS* as a biomarker is unknown. This is because only the technical sensitivity of detection can be derived from the method used and the minimum required neoplastic cell fraction required as input, not the actual values for individual samples. Thus, the clinical significance of high versus lower sensitivity, indicating the number of neoplastic clones with or without a *RAS* mutation, is currently unclear. Achievement of higher sensitivity must, therefore, be weighed against the possibility of false positives or the identification of mutations present only in a small minority of the neoplastic cells, reflecting a small tumor clone population with *RAS* mutations, which is of unknown clinical relevance. Additionally, sensitivity depends on the experience of the laboratory, as results for the LOD of Sanger sequencing were found to be similar to allele-specific methods during blind testing [102]. Furthermore, a recent publication suggests that tumors containing <1 % mutant *KRAS* DNA allele-fraction in microdissected samples might still benefit from treatment with anti-EGFR agents [103]. Overall, it is important to take into account histopathological information from the tissue samples used for mutation detection in future clinical studies, to better define cutoffs for sensitivity and clinical response, and to improve the benefit to patients of targeted therapies.

### Timing of testing

Turnaround times for testing remain a key challenge for pathologists and oncologists, and it is important that a strategy is in place to optimize the process, whether this is through stratified reflex testing based on patient risk factors or through logistical management of new or archived tumor samples. In general, European EQA schemes recommend that the process of obtaining a *RAS* test result should take no more than 10 working days [104], and it is important that pathology and oncology centers work together to address barriers that prevent prompt turnaround of test requests. NGS processes generally take more than 2 days to complete, which is the main bottleneck when choosing new technology in comparison to conventional methods.

Reflex sampling involves the immediate sampling and testing of mCRC at the time of diagnostic biopsy. The major benefit of reflex testing is that information on *RAS* status is available as soon as EGFR-targeted therapies are considered, so no time is lost in starting treatment. Reflex testing in which all colorectal cancers are tested to anticipate the risk of metastasis is also possible, although this remains an area of debate. Underlying *RAS* mutations appear to occur early in tumor development, and therefore, testing for *RAS* mutations is potentially informative, although the prognostic significance of such information is not known. While delays in obtaining *RAS* test results can affect treatment strategies, the implementation of reflex testing is likely to be associated with a high degree of unnecessary testing. Whatever the timing of testing, it is important that interpretation of results must take into account the patient’s clinical history.

### Bioinformatics

The function of the bioinformatics pipeline with regard to *RAS* analysis is to convert the raw sequencing data into clinically useful information through the use of appropriate software algorithms [105]. Integration of NGS technologies into the clinical setting has the potential to verify suggestive relationships and remove ambiguity more quickly than current approaches. Validation of the output from such algorithms can be achieved through the use of reference sequences, control samples containing specific *RAS* variants, and by comparing the output with that from alternative sequencing platforms. Guidelines issued by the National Collaborative Study of Dutch Genome Diagnostic Laboratories state that data analysis and quality checks can be divided into five main steps: base calling and quality assessment of raw data, mapping of reads against a given reference sequence, enrichment analysis, coverage analysis, and variant calling and annotation [106]. The guidelines further state that minimal quality criteria must be established in each laboratory to determine the overall quality of platform performance, although the specific criteria usually depend on the sequencing platform being used.

The initial *RAS* sequencing report should include all variants that were detected against a stated reference sequence, regardless of their clinical significance, although this information should be used to annotate each variant as far as possible (including whether or not clinical validation has been reported [105]. The report should also include information on the test used, including any important limitations, along with base quality scores and confirmatory testing. Notably, a study of genome sequencing across 30 international groups showed that, while there was generally good consistency across bioinformatics analysis and interpretation, there was a lack of concordance with regard to interpretation, report content, and patient consent procedures [107]. Suggested reasons for differences between groups included limited quality control measures; different read aligners and variant calling pipelines; variations in performance of analysis tools, software, and filters; errors in programming or manual analysis; and poor-quality data [105, 107].

### The role and importance of quality assurance programs

Given the large variety of techniques and the importance of laboratories’ experience, it is essential to test and validate centers’ performance with standardized EQA schemes. Use of CE-marked kits or fully validated methods involves proof that quality is maintained after the implementation of the marker in the laboratory. Nevertheless, EQA validation should be done periodically to obtain assurance about the laboratory’s quality.

Guideline recommendations for a European EQA program to ensure accuracy and proficiency in *KRAS* mutation testing were published in 2008 [42]. A number of EQA programs are now available (Table 6), [42, 108–114] such as the European program established by the European Society of Pathology (ESP) for testing biomarker mutations in colorectal cancer [42] or the German Quality Initiative in Pathology (QuIP) (Jung et al., manuscript in preparation). The European program aims to ensure optimal accuracy and proficiency in biomarker testing in colorectal cancer across all participating countries [115] and is organized in collaboration with a European working group and the Biomedical Quality Assurance Research Unit of the University of Leuven [115]. The program provides recommendations and overviews of laboratory methods, standardized operating procedures, and accreditation criteria relevant for *RAS* mutation testing [42, 115, 116].

**Table 6:** Quality assurance programs for *RAS* testing in colorectal cancer

AIOM-SIAPEC Italian Program for EQA in molecular pathology [112]
College of American Pathologists [108]
European Molecular Genetics Quality Network [110]
European Society for Pathology [42]
Gen&Tiss French Program–French Cancer Institute (INCa)–AFAQAP–GFCO [113, 114]
German Society for Clinical Chemistry and Laboratory Medicine [111]
German Society for Pathology–QuIP (Quality Initiative Pathology)^a^
United Kingdom National External Quality Assessment Service [109]

^a^Jung et al. manuscript in preparation

The Organization for Economic Co-operation and Development (OECD) guidelines for Quality Assurance in Molecular Genetic Testing address processes relating to genetic testing for variations in germline DNA sequences that are also applicable to somatic DNA testing. The guidelines cover general principles and best practices, EQA systems, proficiency testing (monitoring the quality of laboratory performance), quality of result reporting, and education and training standards for laboratory personnel [117].

In March 2012, medical oncologists, pathologists, geneticists, molecular biologists, EQA providers and representatives from pharmaceutical industries developed a guideline to harmonize the standards applied by EQA schemes in molecular pathology. The guideline comprises recommendations on the organization of an EQA scheme, defining the criteria for reference laboratories, requirements for EQA test samples, and the number of samples that are needed for an EQA scheme [118]. All EQA schemes should be developed by an expert group with a representing EQA provider, which is also responsible for all organizational aspects and operation of the scheme according to International Organization for Standardization (ISO) 17043 standards. For each scheme, a team coordinator is responsible for the selection, distribution and receipt of cases, and the analysis and reporting of results. Importantly, the samples used in an EQA scheme should reflect the diagnostic and clinical reality as closely as possible, and the turnaround time, as defined by the EQA provider, should reflect the common clinical situation. For a reliable evaluation, at least 10 samples per laboratory need to be analyzed per year and can be provided in a single batch, or in multiple smaller batches. The limit for poor performance should be defined as a score 18 out of 20, with no false-positive or false-negative results. Finally, EQA providers should be encouraged to make the general reports available in the public domain. The ESP Colon EQA scheme is organized in accordance with these recommendations and with the OECD best practice guidelines [42]. OECD Principles of Good Laboratory Practice state that individual testing facilities should have a documented QA program to ensure that studies performed are in compliance with principles of good laboratory practice [119]. A number of authors have stated that participation in internal QA schemes, as well as external schemes, should be mandatory for diagnostic pathology laboratories [95, 120]. Furthermore, the validation or verification of testing methods is a formal requirement for the accreditation of laboratories according to the major international standard applicable to medical testing laboratories: ISO 15189 [121, 122].

The need for QA schemes was highlighted by recent research carried out under the European QA program [45], in which 27 % of participating laboratories genotyped at least 10 % of samples incorrectly, although <5 % of distributed specimens overall were genotyped incorrectly. Errors included false negatives, false positives, and incorrectly genotyped mutations. In addition, 20 % of laboratories reported a technical error for at least one sample. It is encouraging, however, that the majority of laboratories made no mistakes. There are now sufficient quality-assured laboratories to allow *RAS* testing across Europe, and even a small number of EQA rounds can improve quality to a high level [123].

In the future, as new techniques become routinely available, existing EQA programs will need to evolve to address the issues arising from the use of NGS and other increasingly sensitive techniques.

## Conclusions

Pathologists have a key role to play in ensuring that patients receive optimal and appropriate treatment for mCRC. Up-front tumor *RAS* testing—rather than solely *KRAS* exon 2 testing—is critical for patients with mCRC, as *RAS* mutations predict a lack of response to anti-EGFR therapy [14]. The more extensive use of biomarkers is already included in relevant treatment guidelines from ESMO, the NCCN, and the AIO [38, 39, 41] and is increasingly recognized as an essential component of mCRC diagnosis and work-up. The reference methods are sequencing: initially the Sanger method and subsequently pyrosequencing. More recently, NGS techniques for mutational analysis have been developed, the key methods applied to molecular pathology being parallel semiconductor and reversible termination sequencing. Some CE-approved kits are commercially available, but most apply only to *KRAS*. Only five kits currently cover both *KRAS* and *NRAS* genes, although even these do not yet include all potentially relevant mutations. Further kit development should be more flexible to allow for evolution of new biomarkers. In addition, many laboratories have their own in-house methods for assessing mutational status and have the ability to more rapidly adapt testing to clinically validated biomarkers. The different analysis techniques have differing technical sensitivities for detecting mutations, although the ideal process sensitivity threshold—including that relating to the neoplastic cell burden in the sample—and the value of tests with a very high sensitivity are currently unknown. More comprehensive planning of clinical studies in the future is therefore required.

Appropriate tumor sampling and prompt delivery of material to diagnostic laboratories is critical for accurate and timely *RAS* testing. Processes to ensure that the turnaround time from sample request to *RAS* evaluation is as efficient as possible need to be implemented on a national and center-specific level to ensure that patients have the option to receive the most appropriate treatment.

EQA programs play an important role in ensuring that *RAS* testing is carried out to a high standard, especially when laboratory-developed methods are used. EQA testing should include “difficult” samples that are at the LOD of the assay being used and should also test the entire chain of analysis, as there are many different steps involved, from preparation of the FFPE block and DNA to the actual molecular testing, results, and bioinformatic reports.

Finally, *RAS* testing is still a moving field, and important future directions include the extension of NGS to an increased number of biomarkers and the extension of quantitative methods to include circulating plasma DNA.
